# Unexpected consequences: Spontaneous kidney rupture following heavy lifting

**DOI:** 10.1016/j.eucr.2025.103193

**Published:** 2025-09-01

**Authors:** Yousor Al-Qudiemat, Rehan Nasir Khan, Mishari Al-Mutairi, Fahad Alabdulghani, Abdullatif Al-Terki

**Affiliations:** a Department of Radiology, Al-Amiri Hospital, Kuwait; b Department of Urology, Al-Amiri Hospital, Kuwait; c Department of Radiologist, Al-Amiri Hospital, Kuwait

## Abstract

Spontaneous kidney rupture (SKR) is a rare, life-threatening condition that can be challenging to diagnose, particularly in individuals without prior renal pathology. We report a case of a 49-year-old male bodybuilder who experienced sudden left flank pain during weightlifting. CT imaging revealed a massive left retroperitoneal hematoma and grade III renal trauma. The patient had no medical history but admitted to anabolic steroid use. He was initially managed conservatively but required selective angioembolization. He fully recovered and was discharged nine days later. This case highlights SKR's rare presentation in a healthy individual, potentially triggered by extreme physical exertion.

## 1 Introduction

In the absence of trauma, spontaneous kidney rupture is a rare and often overlooked differential diagnosis in previously healthy patients, leading to a diagnostic dilemma. However rare, this condition represents a serious clinical entity with potentially life-threatening complications requiring prompt medical attention.^1^ Studies indicate that 95 % of spontaneous kidney rupture are secondary to underlying pathologies. In this article, we present a case of spontaneous kidney rupture in a patient with no past medical history who developed severe left plank pain during a session of heavy weightlifting. We explore similar reported cases, potential predisposing conditions, and precipitating events that may contribute to this condition.

## 2 Case presentation

A 49-year-old male competitive bodybuilder presented to the emergency unit with sudden-onset left flank pain during a workout. He denied any medical or surgical history, but reported regular anabolic steroids use. The pain began while performing an inclined bench press with >300 kg, with no trauma or fall. On triage, he was afebrile, normotensive and in sinus rhythm, with no urinary symptoms. On examination, the patient was in severe pain, with no visible hematoma, bruising, or signs of external trauma. However, there was significant left-sided abdominal distension with guarding and rigidity.

**Fig. 1:**
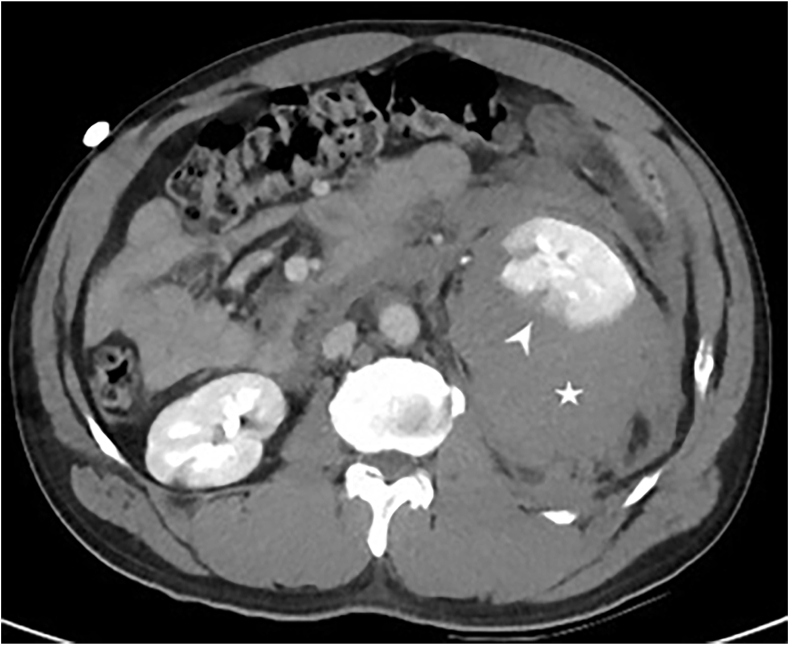
The initial contrast enhanced CT showed a large peri-renal hematoma at the left kidney (star), and a laceration at the lower pole of the kidney (arrow).

Initial investigations showed an elevated serum creatinine of 193 μmol/L (no baseline renal functions available), and a hemoglobin of 172 gm/L. A contrast CT revealed a massive left retroperitoneal hematoma (16.5 x 14.5 × 13.5 cm), displacing and compressing the kidney, with lower pole parenchymal laceration (AAST grade III) (Fig. 1). Three hours later, hemoglobin dropped by 30 g/L, though vitals remained stable. A decision was made for CT angiogram, which revealed a stationary course of the hematoma and renal architecture with no evidence of active bleeding. In hindsight, this drop could likely be explained by the expansion of hematoma. Considering grade III renal injury, with a non-expanding hematoma, the patient was admitted in special care for conservative management with bed rest, serial labs, and analgesia.

**Fig. 2:**
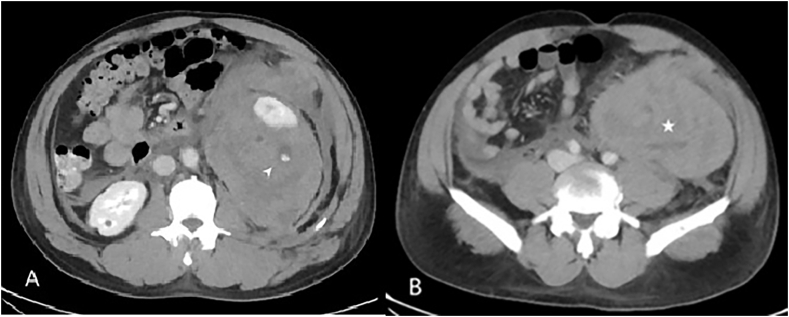
(a) The subsequent CT demonstrates an increase in the size of the hematoma, and arterial contrast extravasation was detected (arrow). (b) The hematoma had expanded and extended into the pelvis.

**Fig. 3:**
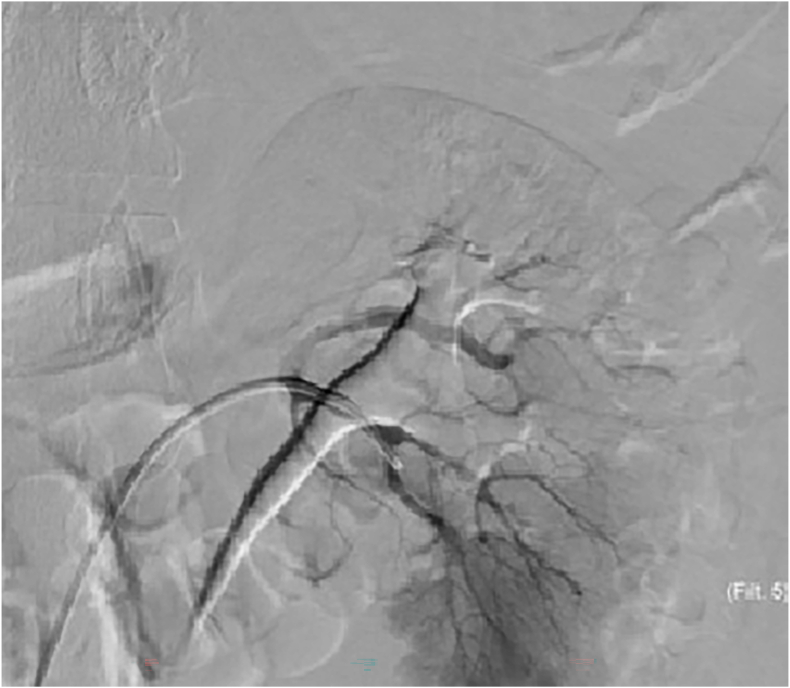
DSA angiography failed to show any contrast extravasation; however, the site of laceration could be seen on the angiographic images.

**Fig. 4:**
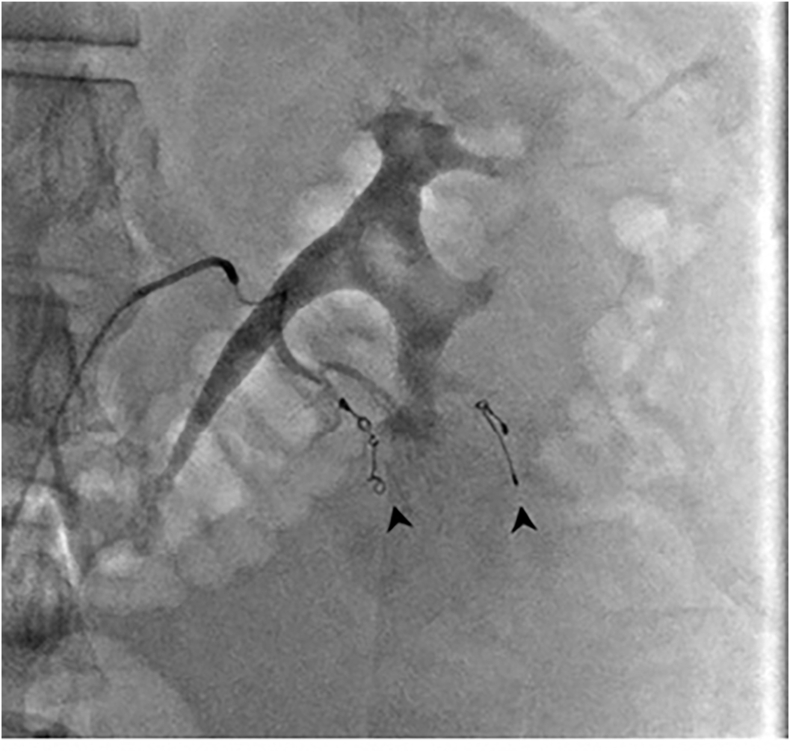
Fluoroscopic screening image showing a 5Fr C2 catheter placed in the left renal artery, and a Progreat 2.7Fr microcatheter in a segmental lower pole artery. Multiple 3mm coils (arrows) have been deployed into peripheral lower pole interlobular renal arteries.

**Fig. 5:**
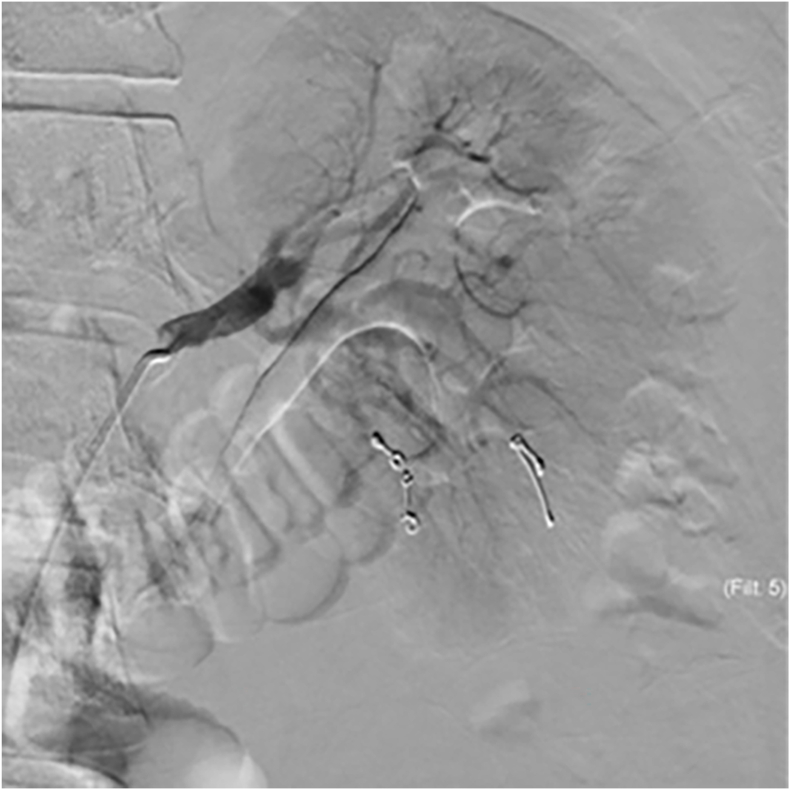
Coils were deployed into interlobular arteries based on the site of laceration and location of extravasation on the previously obtained CT images. DSA post coiling was performed.

Following his admission, he was noted to have persistently elevated blood pressure readings (approximately 160/100 mm Hg), for which he was started on intravenous antihypertensive therapy. After 48 hours, he reported worsening pain and abdominal distension. Blood pressure exceeded 200 mm Hg, and hemoglobin dropped to 8 g/L. Repeat CT angiogram revealed hematoma expansion, further renal compression, and active bleeding from the lower pole (Fig. 2A & B). The patient was promptly shifted to the interventional radiology suite, for angioembolization. Arterial access was obtained in the usual fashion via the right common femoral artery. Digital subtraction angiography was performed, demonstrating lacerations in the renal parenchyma (Fig. 3). The lower pole artery leading to the point of extravasation was identified, and embolized with multiple 3 mm coils (Fig. 4) (Fig. 5).

Two days post-embolization, the patient developed dyspnea, tachypnea and a sudden drop in oxygen saturation to 93 %. Chest x-ray done at the time confirmed the suspicion of fluid overload. Blood work at this point showed a stationary course of hemoglobin, downward trending serum creatinine (176 μmol/L), and negative cardiac markers. He was transferred to the intensive care unit for further evaluation, started on fluid restriction, and supportive management. Repeat CT showed no active bleeding, reduced hematoma size, and improved renal perfusion. By day three, respiratory symptoms resolved, and he was transferred back to special care. His recovery from this point forwards was exemplary. A follow-up CT (day 4 post-embolization) showed no contrast extravasation, stable hematoma (14 x 13 × 16 cm), resolving retro-aortic and peripancreatic extension, decreased free fluid, and preserved renal perfusion. The patient was discharged in stable condition nine days after admission, with outpatient follow-up arranged. At discharge, his laboratory results were satisfactory, with hemoglobin at 11.2 g/L and serum creatinine at 134 μmol/L. However, features consistent with Page kidney phenomenon were noted, contributing to persistently elevated blood pressure, which was managed with antihypertensive therapy.

A low-dose, non-contrast CT was performed one-month post-embolization due to ongoing renal impairment, precluding contrast use. The scan showed the retroperitoneal hematoma remained but had slightly decreased in size (from 14 × 25 × 19 cm to 13 × 25 × 15 cm), extending from L2 to the small pelvis, reaching the iliopsoas muscle. No new complications were identified.

Clinically, the patient remained asymptomatic, with gradually improving renal function despite mild residual impairment. Antihypertensive therapy was continued to manage lingering Page kidney physiology. Given the slow but steady resolution of the hematoma, he was advised to continue blood pressure and renal function monitoring. He was cleared to resume light weightlifting under strict precautions, with a gradual return to his regular training regimen.

## 3 Discussion

Spontaneous kidney rupture (SKR), also known as traumatic rupture of the kidney, was initially described in 1856 by Wunderlich and referred to as “spontaneous apoplexy of the renal capsule”. It represents a rare yet critical condition, in patients presenting with sudden onset flank pain. SKR typically arises from a preexisting, underlying kidney pathology; including congenital causes, obstructive causes, along with other causes such as chronic hemodialysis, renal tumors, renal allograft, and arteriovenous malformations.^2^

A comprehensive understanding of the clinical manifestations and risk factors associated with [spontaneous kidney rupture] is, thus, crucial for making a timely diagnosis and thereby providing the patient with the best chance for a positive health outcome. One of the most extensive series investigating the common etiologies of SKR was described by Zhang and colleagues in a meta-analysis study. Their research revealed that 61 % of 165 cases were attributed to tumors, 17 % to vascular pathologies, and 12.7 % to miscellaneous causes. Similarly, McDougal's study reviewed 78 cases and found that 57.7 % were tumor-related including benign tumors such as angiomyolipoma (9 cases) and lipoma (3 cases), and malignant tumors such as clear cell carcinoma (13 cases) and papillary carcinoma (4 cases). Vascular causes accounted for 17.9 %, with periarteritis nodosa being the most common (10 cases). Infections represented 10.3 %, and other causes included nephritis (5.1 %), blood dyscrasias (5.1 %), and miscellaneous findings (3.9 %).^3^^,^^4^ Furthermore, a numerous number of publications brings to our attention that chronic hemodialysis and kidney transplant could also be a contributing risk for SKR.^5^^,^^6^

Upon further literature review, two cases of SKR preceded by physical activity had been identified. The first case, reported by Yavuzsan et al., involved a 20-year-old male presenting with sudden onset abdominal pain during heavy lifting. The patient complained of a bursting sensation followed by severe pain in the flank region, after-ward the patient underwent a simple left nephrectomy and the urologist had concluded that the kidney was ruptured due to severe hydronephrosis.^7^ The second case reported by Neymark et al. presents a female with ASKR after physical exercise, the patient underwent prompt nephrectomy and was diagnosed with clear cell adenocarcinoma.^8^

What sets our case apart is its absence of the afore mentioned risks. The only significant data from the patient's medical history included several days of intense abdominal-targeted exercise and steroid use, leading us to believe that vigorous exercise directly caused the spontaneous kidney rupture in this instance. Elaborating on the exercise regimen, the patient engaged in a high-repetition, heavy-weight abdominal program involving 300 weighted abdominal crunches up to 120kg over three consecutive days. Despite feeling a popping sensation and mild pain in the left flank on the third day, the patient continued the program, which culminated in exacerbated pain during chest presses the following day, prompting immediate medical attention.

Expanding our discussion to exercise related intra-abdominal injuries, though unrelated to the kidneys, a case study involving a 37-year-old male with no known past medical history reported syncopal episodes left lower quadrant pain following CrossFit training, this report concluded that the patient had a ruptured common iliac artery (CIA) in isolation of an abdominal aortic aneurysm and in a normal Caliber CIA.^9^

Additionally, a number of cases have been reported where pregnancy has been identified as a contributing factor to spontaneous kidney rupture, though unrelated to our case, the underlying similarity lies in the heightened abdominal pressure.^10^, ^11^, ^12^ While prior literature has not established a direct causation between increased abdominal pressure and SKR, our extensive literature review leads us to hypothesize that increased abdominal pressure could serve as a trigger for SKR, particularly in cases involving pathological kidneys.

Exploring other potential risk factors, we have further investigated the use of steroid use and SKR. Evidence suggesting the association of steroid use with endothelial dysfunction raises concerns about its potential impact and the likelihood of spontaneous kidney rupture. We have found that endothelial dysfunction is indeed associated with SKR, making steroid use an indirect risk factor for SKR.^13^ Lastly, although our patient is presumably healthy and denied any medical history, we have found multiple readings of high blood pressure during our patient's hospital stay. This persuades us to believe that the patient might have undiagnosed hypertension, which without question could attribute in SKR. Uncontrolled blood pressure can progress to abdominal compartment syndrome, which is congruent with our discussion between the association of atraumatic spontaneous kidney rupture and increased abdominal pressure.

The limited availability of comparable cases impedes our ability to establish a standardized recovery plan for the patient. A study aiming to evaluate a safe protocol for resuming sporting activities after renal trauma in children has yet to reach a consensus due to diversity in injury stages, mechanism of injury and management. Nevertheless, surveys have indicated that patients with low-grade renal injury (AAST I-III) are typically permitted mobilization two weeks post-injury, whereas individuals with high grade-renal trauma (AAST IV-V) resumed mobilization 3–6 weeks after renal injury.

The detailed account of the patient's strenuous exercise regimen underscores how exercise-induced ASKR can manifest in athletes without trauma or underlying renal pathology.^14^ The scarcity of similar cases in the literature emphasizes the unique nature of our case and underscores the need for further exploration in this domain.

## 4 Conclusion

Although a definitive cause could not be established, we postulate—based on clinical context and existing literature—that prolonged anabolic steroid use combined with elevated intra-abdominal pressure during intense exercise may have contributed to the rupture. This case underscores the value of prompt imaging and radiological intervention for timely diagnosis and management.

In summary, we report a rare case of spontaneous kidney rupture (SKR) in a competitive bodybuilder with no underlying renal pathology but a history of anabolic steroid use. The patient presented with acute left flank pain following heavy exertion. This case adds to the limited literature on SKR in otherwise healthy individuals.

Ongoing documentation of such cases may improve recognition of the potential link between high-intensity exercise, performance-enhancing drugs, and spontaneous renal injury—supporting the development of safer training protocols, especially for athletes and those at risk of renal compromise.

## CRediT authorship contribution statement

**Yousor Al-Qudiemat:** Writing – review & editing, Writing – original draft, Resources, Project administration, Methodology, Investigation, Data curation, Conceptualization. **Rehan Nasir Khan:** Writing – review & editing, Writing – original draft, Visualization, Supervision, Methodology, Formal analysis, Data curation, Conceptualization. **Mishari Al-Mutairi:** Investigation, Data curation. **Fahad Alabdulghani:** Writing – review & editing, Supervision, Software, Project administration. **Abdullatif Al-Terki:** Supervision, Project administration.

## References

[1] Yoon J.C., Kim W., Cho G.C. (2001 Dec 1). Idiopathic spontaneous renal rupture. Journal of The Korean Society of Emergency Medicine.

[2] Bosniak M.A. (1989 Sep). Spontaneous subcapsular and perirenal hematomas. Radiology.

[3] Zhang J.Q., Fielding J.R., Zou K.H. (2002 Apr 1). Etiology of spontaneous perirenal hemorrhage: a meta-analysis. J Urol.

[4] Koo V., Duggan B., Lennon G. (2004 May). Spontaneous rupture of kidney with peri-renal haematoma: a conservative approach. Ulst Med J.

[5] Cavoli G.L., Bono L., Tortorici C. (2012 Mar 1). Spontaneous kidney rupture in a patient on chronic hemodialysis. Indian J Nephrol.

[6] Lee S., Jung S., Kim H.J. (2021 May 21). Spontaneous rupture of a renal artery pseudoaneurysm in a hemodialysis patient: a case report. Medicine.

[7] Yavuzsan A.H., Baloğlu I.H., Albayrak A.T., Bursali K., Demirel H.C., Demirel C.H. (2021 May 30). Spontaneous kidney rupture: two case reports with unusual presentations. Cureus.

[8] Neymark A.I., Neymark B.A., Nozdrachev N.A. (2022 Feb). Spontaneous rupture of kidney tumor. Successful surgical treatment. Urologiia.

[9] Eguez C., Zhang J. (2022 Sep 16). Spontaneous common iliac artery rupture due to high intensity exercise. Cureus Journal of Medical Science.

[10] Cardenas R.T., Doiron T.E., Ramseyer A.M., Pates J.A., Po W.D., Magann E.F. (2021 Sep 1). Spontaneous renal rupture during pregnancy: a contemporary literature review and guide to management. Obstetrical & Gynecological Survey.

[11] Middleton Jr AW., Middleton G.W., Dean L.K. (1980 Jan 1). Spontaneous renal rupture in pregnancy. Urology.

[12] Oesterling J.E., Besinger R.E., Brendler C.B. (1988 Sep 1). Spontaneous rupture of the renal collecting system during pregnancy: successful management with a temporary ureteral catheter. J Urol.

[13] Knight S.F., Quigley J.E., Yuan J., Roy S.S., Elmarakby A., Imig J.D. (2008 Feb 1). Endothelial dysfunction and the development of renal injury in spontaneously hypertensive rats fed a high-fat diet. Hypertension.

[14] Valtonen E.J. (1966 Oct). Spontaneous rupture of an apparently normal kidney; some criticism concerning this diagnosis. BJU.

